# An Epilepsy-Associated Mutation of Salt-Inducible Kinase 1 Increases the Susceptibility to Epileptic Seizures and Interferes with Adrenocorticotropic Hormone Therapy for Infantile Spasms in Mice

**DOI:** 10.3390/ijms23147927

**Published:** 2022-07-18

**Authors:** Bo Pang, Takuma Mori, Moataz Badawi, Mengyun Zhou, Qi Guo, Emi Suzuki-Kouyama, Toru Yanagawa, Yoshinori Shirai, Katsuhiko Tabuchi

**Affiliations:** 1 Department of Molecular and Cellular Physiology, Shinshu University School of Medicine, Matsumoto 390-8621, Nagano, Japan; 16mh081k@shinshu-u.ac.jp (B.P.); 20hm124d@shinshu-u.ac.jp (M.Z.); 18hm181d@shinshu-u.ac.jp (Q.G.); emi_suzuki@shinshu-u.ac.jp (E.S.-K.); yoshirai@shinshu-u.ac.jp (Y.S.); 2 Department of NeuroHealth Innovation, Institute for Biomedical Sciences, Interdisciplinary Cluster for Cutting Edge Research, Shinshu University, Matsumoto 390-8621, Nagano, Japan; 3 Strathclyde Institute of Pharmacy and Biomedical Sciences, University of Strathclyde, 161 Cathedral Street, Glasgow G4 0RE, UK; mabdallab@gmail.com; 4 Department of Oral and Maxillofacial Surgery, Faculty of Medicine, University of Tsukuba, Tsukuba 305-8575, Ibaraki, Japan; ytony@md.tsukuba.ac.jp

**Keywords:** SIK1, DEE-30, infantile spasms, ACTH, NMDA

## Abstract

Six mutations in the salt-inducible kinase 1 (SIK1) have been identified in developmental and epileptic encephalopathy (DEE-30) patients, and two of the mutations are nonsense mutations that truncate the C-terminal region of SIK1. In a previous study, we generated SIK1 mutant (SIK1-MT) mice recapitulating the C-terminal truncated mutations using CRISPR/Cas9-mediated genome editing and found an increase in excitatory synaptic transmission and enhancement of neural excitability in neocortical neurons in SIK1-MT mice. NMDA was injected into SIK1-MT males to induce epileptic seizures in the mice. The severity of the NMDA-induced seizures was estimated by the latency and the number of tail flickering and hyperflexion. Activated brain regions were evaluated by immunohistochemistry against c-fos, Iba1, and GFAP. As another epilepsy model, pentylenetetrazol was injected into the adult SIK1 mutant mice. Seizure susceptibility induced by both NMDA and PTZ was enhanced in SIK1-MT mice. Brain regions including the thalamus and hypothalamus were strongly activated in NMDA-induced seizures. The epilepsy-associated mutation of SIK1 canceled the pharmacological effects of the ACTH treatment on NMDA-induced seizures. These results suggest that SIK1 may be involved in the neuropathological mechanisms of NMDA-induced spasms and the pharmacological mechanism of ACTH treatment.

## 1. Introduction

Salt-inducible kinase 1 (SIK1) is an AMP-activated protein kinase (AMPK) inducibly expressed in the adrenal cortex in response to the salt intake or adrenocorticotropic hormone (ACTH) [1,2,3,4,5]. SIK1 has been shown to play an essential role in the ACTH-signaling pathway. SIK1 mRNA expression in cultured adrenal cortical cells is induced and peaks in 1 h after the application of ACTH [2]. SIK1 has been shown to modulate ACTH signaling. ACTH acts on the melanocortin receptor 2, a Gs type G protein-coupled receptor, resulting in the induction of cAMP followed by the activation of cAMP-dependent protein kinase (PKA) [4]. PKA phosphorylates a serine at position 577 (S577) of SIK1 [6,7]. Phosphorylated SIK1 upregulates the cAMP response element binding (CREB) protein and downregulates the myocyte enhancer factor 2 (MEF2) functions by phosphorylating cAMP-regulated transcriptional co-activators (CRTC) and the class IIa histone deacetylases (HDAC), respectively [8,9].

SIK1 is also detected in the brain regions, which include the cerebral cortex and hippocampus [10,11,12,13]. In 2015, Hansen et al. reported six SIK1 mutations in developmental and epileptic encephalopathy (DEE)-30 (OMIM # 616341, aka EIEE-30), manifesting myoclonic encephalopathy, Ohtahara syndrome, or infantile spasms [14]. Two out of six patients had nonsense mutations, resulting in the truncation of the C-terminal of the SIK1 protein and display infantile spasms and developmental abnormalities, including absent speech, repetitive behaviors, and poor social interactions. Infantile spasms occur one incident in three thousand births, and ACTH is used as a first-line treatment of infantile spasms. One of the two patients was a female (IS13-013). She was initially treated with phenobarbital but later with ACTH. Both phenobarbital and ACTH were ineffective for her symptoms. This report raises a possibility that SIK1 is involved in ACTH-treatment of the infantile spasms.

We previously generated SIK1 mutant (SIK1-MT) mice recapitulating the C-terminal truncated mutation of SIK1 using CRISPR/Cas9-mediated genome editing as a disease model of the human cases [15]. Heterozygote SIK1-MT mice exhibited autistic behaviors, including repetitive behaviors and impaired social interactions, as seen in human patients. The excitability of neurons and excitatory synaptic transmission in the medial prefrontal cortex were increased in SIK1-MT mice. The administration of risperidone ameliorated the excitability of neurons, excitatory synaptic transmission, and repetitive behavior but failed to restore the social behavior in SIK1-MT mice. Risperidone also attenuated the inhibitory synaptic transmission in SIK1-MT mice. This hampered the correction of excitatory and inhibitory synaptic balance, even after the excitatory synaptic transmission became normal. The impaired excitatory and inhibitory balance may be the cause of the impaired social behavior in SIK1-MT mice.

Although the relevance between the SIK1 mutation and autistic behaviors has been uncovered in SIK1-MT mice, the effect of the SIK1 mutation on epilepsy remains enigmatic. In addition, considering the role of SIK1 in ACTH signaling, it would be intriguing to investigate the effect of ACTH on SIK1-MT mice because ACTH is the first-line drug for infantile spasms [16,17,18]. In the present study, we investigated the effect of the C-terminal truncated mutation of SIK1 on epilepsy by inducing seizures in SIK1-MT mice. We applied N-methyl-D-aspartate (NMDA) to juvenile SIK1-MT mice to induce spasms and investigated the severity of spasms and the effect of ACTH on the spasms. We also studied the susceptibility to temporal lobe epilepsy in SIK1-MT mice by inducing seizures with pentylenetetrazol (PTZ) in adult mice. SIK1-MT mice displayed an enhanced seizure susceptibility induced by both NMDA and PTZ. SIK1-MT mice did not respond to ACTH for NMDA-induced spasms. These results suggest that SIK1 mutation may be the cause of epilepsy and the dysfunction of the SIK1 results in the resistance to ACTH treatment in DEE patients.

## 2. Results

### 2.1. NMDA-Induced Spasms in Wild-Type and SIK1 Mutant Mice

**Figure 1:**
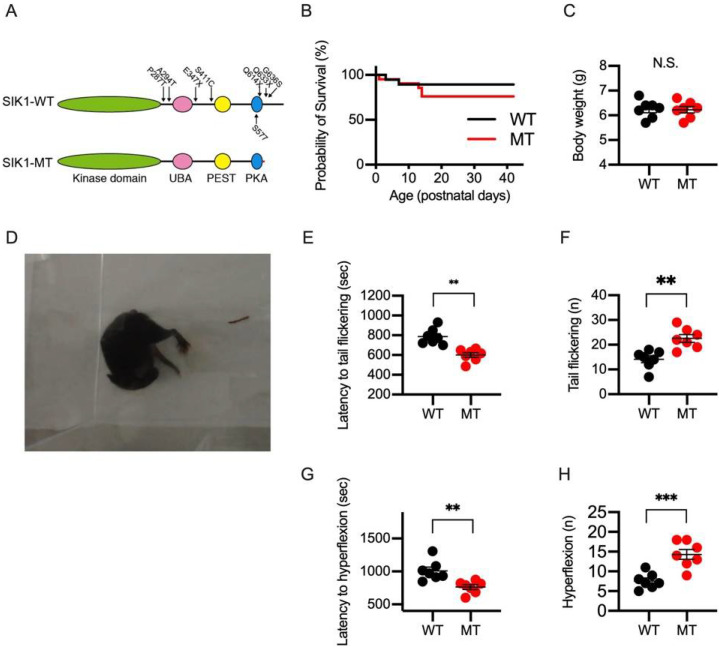
NMDA-induced seizures in wild-type and SIK1 mutant mice. (**A**) Schematic diagram of the protein structure of SIK1 in wildtype (WT, top) and mutant (MT, bottom) mice used in this study. Kinase domain (green), ubiquitin-associated (UBA) domain (pink), proline-glutamate-serine-threonine (PEST, yellow) domain, and protein kinase A target domain (PKA, blue) are indicated. Epilepsy-associated mutations are indicated by arrows. Truncated C-terminal in SIK1-MT mice is indicated in red. (**B**) Survival rates of the wild-type (n = 19) and SIK1-MT (n = 21) mice. Significant lethality is not observed in SIK1-MT mice. (**C**) Body weight of the wild-type and SIK1-MT mice. Significant difference in body weight is not detected in SIK1-MT mice. (**D**) Representative photo image of mouse posture during NMDA-induced spasms. See also supplementary movie S1. (**E**) Summary graph for onset of tail flickering after NMDA injection. The onset of tail flickering is shortened in SIK1-MT mice. The number on the bar indicates that of mice used in each experimental condition. (**F**) Summary graph for total number of tail flickering in NMDA-injected wild-type and SIK1-MT mice. Total number of tail flickering is 1.5 times higher than in wild-type mice. (**G**) Summary graph for onset of hyperflexions after NMDA injection. The onset of hyperflexions is shortened in SIK1-MI mice. (**H**) Summary graph for total number of hyperflexions after NMDA injection. The number of hyperflexions is increased in SIK1-MT mice. Data are described in a scatter plot of each value with means ± SEM. No statistical difference was detected by log-rank (Mantel-Cox) nor Gehan–Breslow–Wilcoxon tests (**B**). Statistical analyses (in **C**,**E**–**H**) were performed by Student’s *t*-test (** *p* < 0.01; *** *p* < 0.005; N.S. = not significant).

Truncations of the SIK1 gene in two patients with infantile spasms occurred within a nuclear localization domain (NLD) in the C-terminal region [14]. We previously established a mouse line with a frame-shift mutation in the SIK1 gene that produces a truncated form of SIK1 protein and studied heterozygote SIK1-MT mice as models for human patients (Figure 1A) [15]. In this study, we analyzed male heterozygote SIK1-MT mice as models for DEE-30 and, hereafter, referred to them as SIK1-MT mice.

SIK1-MT mice were viable without showing a significant reduction in the survival rate (Figure 1B). No growth abnormalities were shown in SIK1-MT mice, as we reported previously (Figure 1C). Obvious spontaneous spasms or seizures were undetected during our daily observation in SIK1-MT mice. To assess the relevance of the SIK1-MT to the pathophysiology of infantile spasms, we applied provocative drugs to induce seizures in SIK1-MT mice. NMDA has been shown to induce infantile spasms in juvenile rodents [19,20]. We intraperitoneally administered NMDA to postnatal 13-day-old (P13), juvenile, wild-type, and SIK1-MT mice (5 mg/kg, i.p.) and observed seizure-like behaviors for 40 min. Injection of NMDA induced tail flickering at 10 min (Supplementary Movie S1), and hyperflexions at 15 min (Figure 1C and Supplementary Movie S2). Spasm-like seizures with symmetrical flexor and extensor spasms of the whole body were observed in some mice (Supplementary Movie S3). The latency to tail flickering was longer in SIK1-MT (798.8 ± 33.8 s) mice compared to that of wild-type mice (592.3 ± 25.9 s, *p* = 0.0007 in Student’s *t*-test) (Figure 1D). The total number of tail flickering was increased in SIK1-MT (23.2 ± 1.7) compared to that in the wild-type mice (15.3 ± 0.9, *p* = 0.0022 in Student’s *t*-test) (Figure 1E). In our experimental condition, hyperflexion was developed after the tail flickering. The latency to the hyperflexion was shorter, and the total number of the hyperflexions was increased in SIK1-MT mice compared to those in wild-type mice (Figure 1F,G). All the SIK1-MT mice died within 1 h after the first tail flickering, while only a part of the wild-type mice group (1/7 mice at 10 mg NMDA/kg BW) died by NMDA injection. This indicates NMDA-induced spasms are more severe in the SIK1-MT than in the wild-type mice.

### 2.2. Brain Regions Activated during NMDA-Induced Spasms

**Figure 2:**
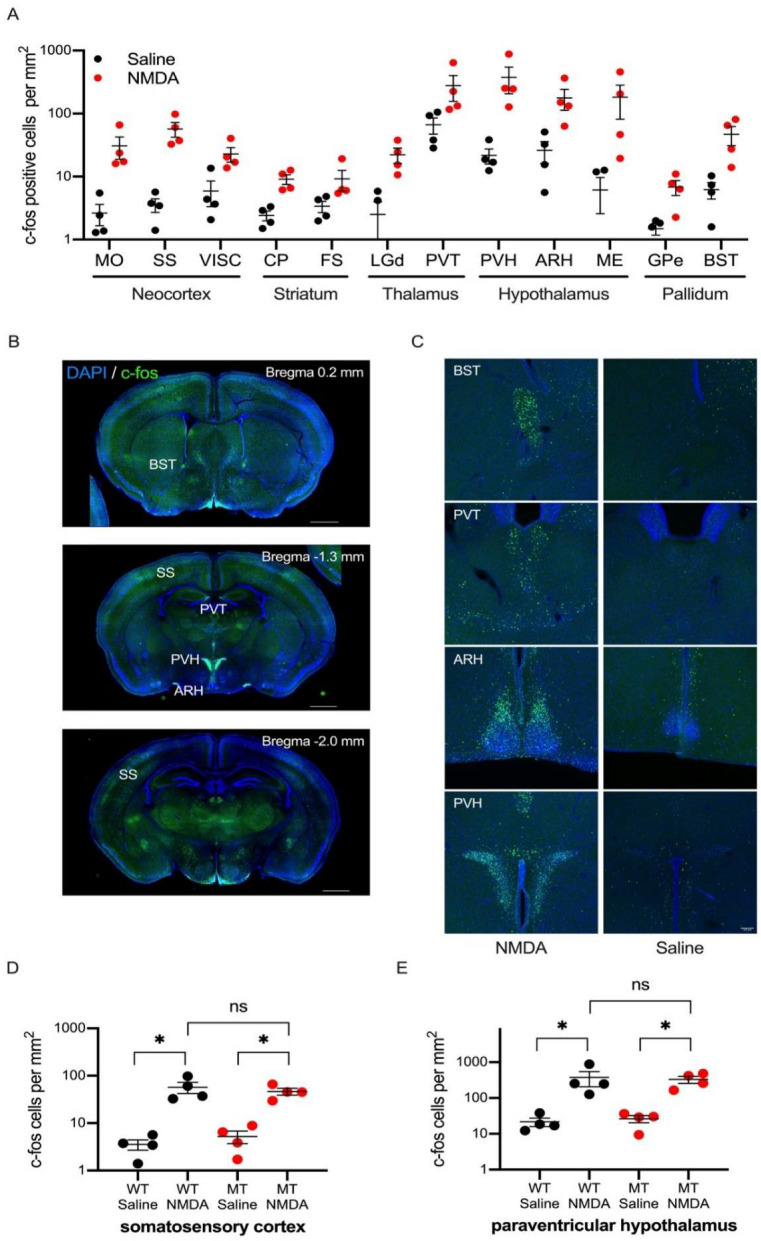
c-fos expression after NMDA-induced seizure in wild-type mice. (**A**) Density of c-fos positive neurons across different brain regions in saline- (black) or NMDA- (red) injected mice are depicted in the graph. Brain regions which exhibited a significant increase in the c-fos positive neurons are shown in the graph. Full data is available in Supplementary Table S1. Abbreviations (MO: somatomotor areas, SS: somatosensory areas, VISC: visceral area, CP: caudoputamen, FS: fundus of striatum, LGd: dorsal part of lateral geniculate complex, PVT: paraventricular nucleus of thalamus, PVH: paraventricular hypothalamic nucleus, ARH: arcuate hypothalamic nucleus, ME: median eminence, GPe: globus pallidus, external segment, BST: bed nucleus of stria terminalis). (**B**) Representative images of c-fos immunostaining in NMDA-injected wild-type mouse brain sections. Scale bar = 1 mm. (**C**) Representative images of c-fos immunostaining in NMDA- (left) and saline- (right) injected wild-type mouse brain sections. Brain images, including BST, PVT, ARH, and PVH, are shown (from top to bottom). (**D**) Density of c-fos positive neurons in the somatosensory cortex (SS) in saline- or NMDA-injected wildtype (WT) or SIK1-MT (MT) mice. (**E**) Density of c-fos positive neurons in the paraventricular hypothalamic nucleus (PVH) in saline- or NMDA-injected wildtype (WT) or SIK1-MT (MT) mice. Data are described in a scatter plot of each value with means ± SEM. Significant differences were detected by ANOVA Bonferroni post hoc test (* *p* < 0.05; ns, not significant). Scale bar = 1 mm (**B**) or 100 μm (**C**).

To identify the activated brain regions during NMDA-induced spasms, we analyzed the expression of an immediate–early gene, c-fos. Brain sections of NMDA and saline-injected mice were stained with the anti-c-fos antibody and analyzed for the distribution of c-fos positive cells in 126 brain regions defined by the Allen Brain Atlas (Supplementary Table S1). Since all the SIK1-MT mice died shortly after the NMDA injection, we compared saline or NMDA-injected wild-type animals. We found a significant increase in the density of c-fos positive cells in 12 out of 126 brain regions (Figure 2 and Supplementary Table S1). The activated brain regions include the neocortex (MO, SS, and VISC), striatum (CP and FS), thalamus (LGd and PVT), hypothalamus (PVH, ARH, and ME), and pallidum (GPe and BST). Among these brain regions, c-fos positive cell density was highest in the paraventricular nucleus of the hypothalamus (PVH) after the NMDA-induced spasms. To investigate a possible causality, we analyzed the correlations between c-fos expression and epileptic properties, but we did not observe a positive or negative correlation between c-fos expression and epileptic characteristics (Supplementary Figure S1). Furthermore, we compared c-fos expression levels in SS and PVH in wild-type and SIK1-MT mice treated with saline or NMDA (5 mg/kg). We found that NMDA treatment upregulated c-fos expression in both wild-type and SIK1-MT mice (Figure 2D,E). The c-fos positive cell density was not different between the wild-type and SIK1-MT mice.

**Figure 3:**
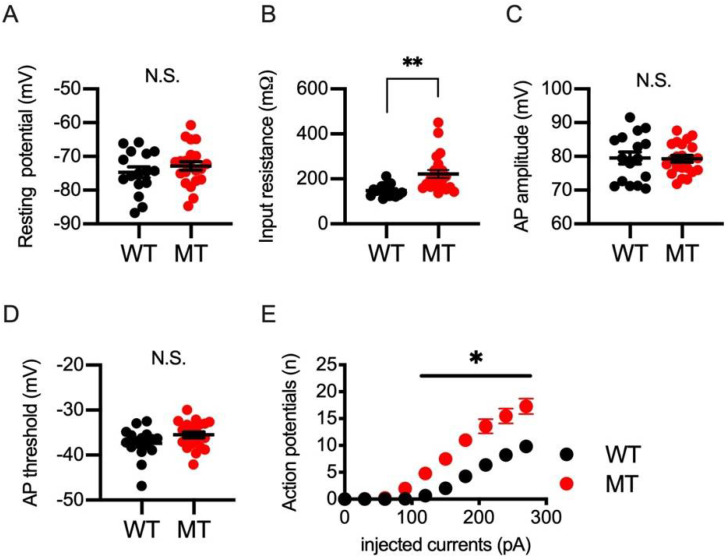
Electrophysiological parameters of neurons in the somatosensory cortex of wild-type and SIK1-MT mice. (**A**) Resting potential was unchanged between wild-type and SIK1-MT mice. (**B**) Input resistance of neurons was increased in SIK1-MT more than in wild-type mice. (**C**) The amplitude or (**D**) the threshold of the action potential of neurons was not changed between both genotypes. (**E**) The number of action potentials by injected currents was increased in SIK1-MT mice more than in wild-type animals. Data are described in a scatter plot of each value with means ± SEM. Significant difference was examined by Student’s *t*-test (* *p* < 0.05; ** *p* < 0.01; N.S. = not significant).

To examine whether neuronal excitability was altered in SIK1-MT mice, we analyzed the electrophysiological properties of the cortical neurons in SS using patch clamp recordings. We measured the resting potential (Figure 3A), the input resistance (Figure 3B), the amplitude of the action potentials (Figure 3C), and the threshold of the action potentials (Figure 3D). We found that input resistance was increased in SIK1-MT mice, indicating a tendency to increase neuronal excitability. We also measured the number of action potentials induced by injected electrical currents and found that more action potentials were evoked by the injection of electrical currents (Figure 3E).

**Figure 4:**
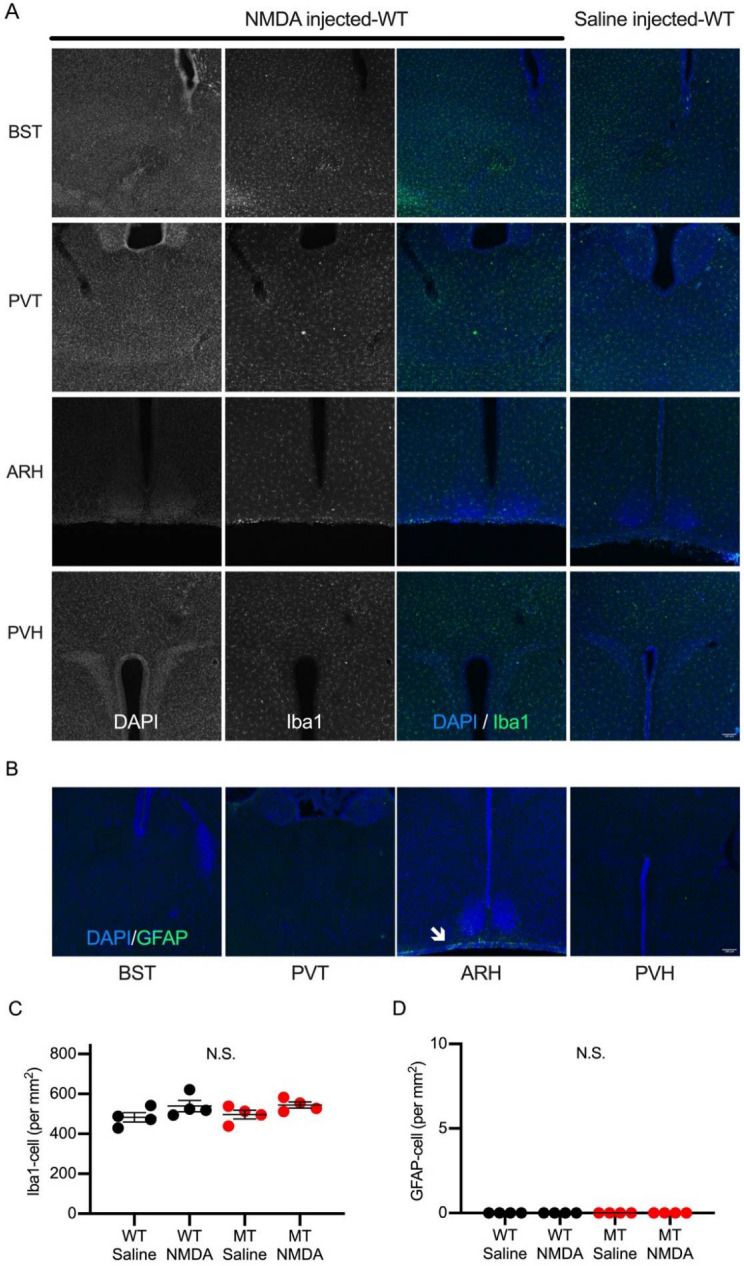
Iba1 and GFAP immunoreactivity in NMDA or saline-injected wild-type mouse brain sections. (**A**) Confocal images of Iba1 immunopositive microglia on brain regions (from top, BST, PVT, ARH, and PVH) are shown. No significant difference in Iba1 expression patterns were observed between NMDA- (left) and saline- (right) injected mice. Scale bar = 100 μm. (**B**) Confocal images of GFAP-immunostained brain sections (from left, BST, PVT, ARH, and PVH) in NMDA-injected mice are shown. GFAP signals (arrow) were observed only around the pia mater in the basal part of the hypothalamus. (**C**) Density of Iba1 positive microglia in PVH was not changed in genotypes or treatments. (**D**) Density of GFAP positive astrocytes in PVH was not changed in genotypes or treatments. No GFAP signals were observed in either of the mice. Data are described in a scatter plot of each value with means ± SEM. Significant difference was examined by ANOVA Bonferroni post hoc test (N.S., not significant). Scale bar = 100 μm.

As well as the c-fos expression, brain inflammation, such as the transformation of microglia and astrocytes, has been shown to accompany many cases of seizures [21,22]. We visualized microglia by immunohistochemistry against Iba1, a molecular marker for microglia (Figure 4A), and GFAP (Figure 4B), a marker for astrocytes. We then investigated the distributions of microglia and astrocytes in brain regions in saline or NMDA-injected animals. The immunoreactivity to Iba1 was unchanged in PVT, PVH, and somatosensory cortex in NMDA-injected animals compared to saline-injected animals. We calculated the density of Iba1 immunopositive cells in PVH, and no change was detected between each genotype and treatment (Figure 4C). The immunoreactive signal for GFAP was faint in P13 mice, probably because astrocytes develop at a later stage in brain maturation. We did not find any GFAP positive cells in PVH in all the conditions (Figure 4D).

### 2.3. Effects of ACTH on NMDA Spasms in Wild-Type and SIK1 Mutant Mice

**Figure 5:**
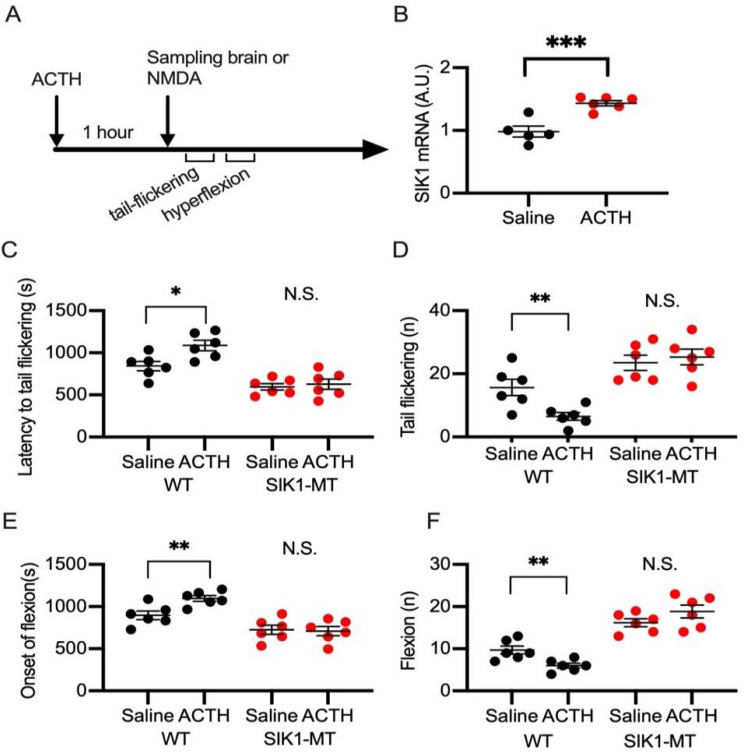
Pretreatment with ACTH is ineffective in NMDA-induced seizures in SIK1-MT mice. (**A**), Time course of ACTH pretreatment in NMDA-induced seizures. (**B**) Levels of SIK1 mRNA estimated by RT-qPCR. ACTH pretreatment increased the levels of SIK1 mRNA in the brain. (**C**) Summary graph for latency to tail flickering with pretreatment of saline or ACTH in wild-type and SIK1-MT mice. Decreased latency to tail flickering in SIK1-MT mice was not recovered by pretreatment with ACTH. (**D**) Summary graph for number of tail flickerings with pretreatment of saline or ACTH in wild-type and SIK1-MT mice. Increased number of tail flickerings in SIK1-MT mice was not recovered by pretreatment with ACTH. (**E**) Summary graph for latency to latency to flexion with pretreatment of saline or ACTH in wild-type and SIK1-MT mice. Decreased latency to flexion in SIK1-MT mice was not recovered by pretreatment with ACTH. (**F**) Summary graph for number of hyperflexions with pretreatment of saline or ACTH in wild-type and SIK1-MT mice. Increased number of hyperflexions in SIK1-MT mice was not recovered by pretreatment with ACTH. Data are described in a scatter plot of each value with means ± SEM. Statistical analyses were performed by Student’s *t*-test (* *p* < 0.05; ** *p* < 0.01; *** *p* < 0.005; N.S. = not significant).

Injection of ACTH has been established as a first-choice therapy for patients with infantile spasms. Pretreatment of ACTH in juvenile rodents has been reported to ameliorate the spasms induced by NMDA injection [20,23,24]. To examine the effect of ACTH on NMDA-induced spasms in SIK1-MT mice, we injected ACTH an hour before the NMDA injection to wild-type and SIK1-MT mice (Figure 5A). We next measured the level of SIK1 mRNA by ACTH pretreatment and found that ACTH administration increased SIK1 mRNA by 1.46 fold (0.9817 ± 0.0865 in saline vs. 1.4360 ± 0.04219 in ACTH) (Figure 5B). Consistent with the previous reports, we observed that ACTH pretreatment prolonged the latency to the first tail flickering (Figure 4A) and flexion (Figure 4B) and reduced the numbers of tail flickerings (Figure 4C) and flexions (Figure 4D) in wild-type mice. Pretreatment of ACTH, however, did not alleviate the severity of NMDA-induced spasms evaluated by these parameters in SIK1-MT mice (Figure 4A–D). These results indicate that the normal function of SIK1 may be required for the mechanism alleviating spasms by ACTH treatment.

### 2.4. PTZ-Induced Seizures in Wild-Type and SIK1 Mutant Mice

**Figure 6:**
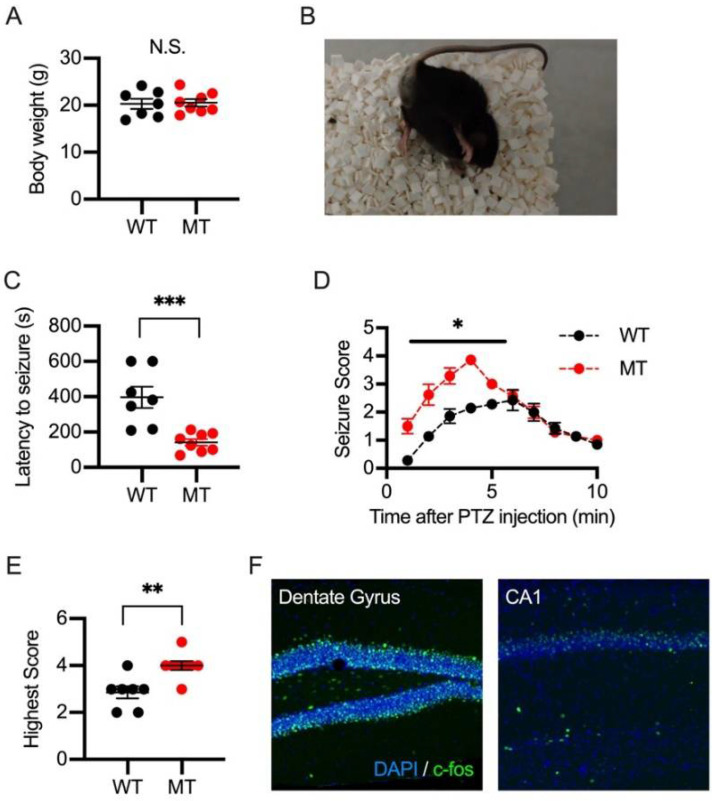
PTZ-induced seizures in wild-type and SIK1 mutant mice. (**A**) Body weight of the 6-week-old wild-type and SIK1-MT mice. Body weight of 6-week-old mice was unchanged. (**B**) Representative photo image of mouse posture during PTZ-induced spasms. See also Supplementary Movie S4. (**C**) Summary graph for latency to seizure in PTZ-injected wild-type and SIK1-MT mice. Latency to seizure was decreased in SIK1-MT mice. (**D**) Summary graph for time course of seizure score after PTZ injection. The seizure score is significantly increased in SIK1-MT mice 3–5 min after PTZ injection. (**E**) Summary graph for highest seizure score in wild-type and SIK1-MT mice throughout the time course after PTZ injection. (**F**) Representative images for c-fos immunostaining in wild-type and SIK1-MT brain sections after PTZ injection. Scale bar = 100 μm. Data are described in a scatter plot of each value with means ± SEM (numbers of animals examined are shown in graphs). Statistical analyses were performed by Student’s *t*-test (* *p* < 0.05; ** *p* < 0.01; *** *p* < 0.005; N.S. = not significant).

We previously found that the excitability and synaptic function of excitatory neurons were enhanced in the cerebral cortex of SIK1-MT mice. The enhanced excitatory neural activity is a common cause of epilepsy. We next examined whether SIK1 is also involved in temporal lobe epilepsy, a major type of epilepsy in adulthood. The body sizes in adulthood are identical between the wild-type and SIK1-MT mice, indicating that the SIK1 mutation did not affect the growth of the mouse (Figure 6A). Intraperitoneal injection of PTZ has been used to induce seizures that replicate temporal lobe epilepsy in rodents (Figure 6B and Supplementary Movie S4). The SIK1-MT mice developed the first seizure in a shorter latency after PTZ administration than wild-type mice (Figure 6C). The seizure score in the SIK1-MT mice is higher 3–5 min after PTZ injection compared to that in the wild-type mice (Figure 6D). The highest score throughout total observation time (10 min) is higher in the SIK1-MT mice (4.0 ± 0.19) than in the wild-type mice (2.9 ± 0.26). (Figure 6E). In the PTZ-induced seizure model, c-fos expression was prominent in the dentate gyrus and CA1 region of the hippocampus (Figure 6F).

### 2.5. Laminar Distribution of c-fos Positive Cells in the Somatosensory Cortex in PTZ and NMDA-Injected Mice

**Figure 7:**
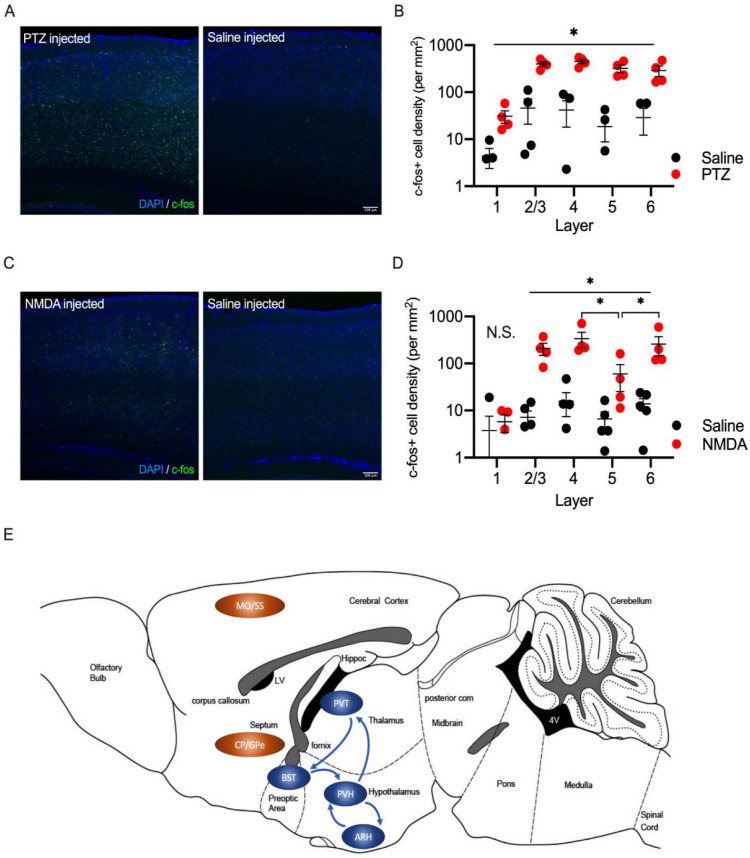
Laminar distribution of c-fos expression after PTZ or NMDA injection in wild-type mice. (**A**) Representative images for c-fos (green) immunostaining in the somatosensory region of wild-type mice after PTZ (left) or saline (right) injection. Scale bar = 100 μm. (**B**) Summary graph for layer distribution of c-fos positive neurons in wild-type mice after PTZ or saline injection. (**C**) Representative images for c-fos immunostaining in the somatosensory region of wild-type mice after NMDA or saline injection. Scale bar = 100 μm. (**D**) Summary graph for layer distribution of c-fos positive neurons in wild-type mice after NMDA or saline injection. No significant difference was detected in layer 1. The density of c-fos positive cells was increased in the other cortical layers. The density of c-fos cells in NMDA-injected mice was smaller in layer 5 than in layers 4 and 6. (**E**) Activated brain regions after NMDA injection are summarized in a cartoon. Sensorimotor regions are indicated in orange. Stress associated regions are indicated in blue. Arrows indicate known neural connections. Abbreviations (MO: somatomotor areas, SS: somatosensory areas, CP: caudoputamen, FS: fundus of striatum, PVT: paraventricular nucleus of thalamus, PVH: paraventricular hypothalamic nucleus, ARH: arcuate hypothalamic nucleus, GPe: globus pallidus, external segment, BST: bed nucleus of stria terminalis). Data are described in a scatter plot of each value with means ± SEM (numbers of animals examined are shown in graphs). Statistical analyses were performed by Student’s *t*-test (* *p* < 0.05; N.S. = not significant).

Both NMDA and PTZ injection also upregulated c-fos expression in the neocortex, such as the somatosensory cortex (Figure 7A–D). We analyzed the laminar distribution of c-fos positive neurons in the somatosensory cortex in PTZ (adult: 6-week-old) and NMDA- (juvenile: P13) injected wild-type mice. The density of c-fos positive cells was elevated in all cortical layers in PTZ-injected mice compared to the saline-injected mice (Figure 7B). On the other hand, the density of c-fos positive cells was elevated in layers 2/3, 4, 5, and 6, but not layer 1 in NMDA-injected juvenile mice compared to the saline-injected mice (Figure 7D). The elevation of c-fos positive density in layer 5 was milder in NMDA-injected mice (Figure 7D). These results indicate that the neural circuits activated by PTZ and NMDA injection may be different.

## 3. Discussion

In the present study, we found that (1) seizure susceptibility induced by both NMDA and PTZ was enhanced in SIK1-MT mice, (2) distinct brain regions were activated in NMDA-induced seizures, (3) no microglial activation was detected in NMDA-induced seizure, (4) SIK1-MT canceled the effect of ACTH treatment on NMDA-induced seizure, (5) distinctive neurons within the cortical layer formation were activated in NMDA- or PTZ-induced seizures.

Susceptibility to drug-induced seizures was increased in SIK1-MT mice. Results in the latency to tail flickering, the number of tail flickerings, latency to hyperflexion, and the number of hyperflexions indicate the drug-induced seizures were more severe in SIK1-MT mice. This suggests that C-terminal truncation of SIK1 may be the cause of seizures in human patients [14]. We failed to detect obvious spontaneous seizures in both homozygote and heterozygote SIK1-MT mice. In this study, we did not perform continuous observations, such as 24-h video recording or electroencephalography (EEG). Thus, we cannot completely exclude the possibility that SIK1-MT mice have spontaneous seizure phenotypes, such as human patients [14,25].

NMDA administration has been used to induce infantile spasms in rodent models. We observed NMDA-induced seizures mimicking human infantile spasms in mice. We investigated the c-fos immunoreactivity in response to the NMDA-induced seizure in 126 brain regions. We identified 12 brain regions where c-fos immunoreactivity was significantly increased. The most prominent c-fos upregulation was observed in the paraventricular hypothalamus (PVH). PVH is an area where corticotropin release hormone (CRH) is produced [26,27,28]. CRH is known to be a “stress hormone”, and PVH is considered to be involved in the neural network related to the stress pathway (HPA axis) together with PVT, BST, and ARH, as indicated in the blue color in Figure 6E [29,30,31]. c-fos expression was also upregulated in PVT, BST, and ARH after NMDA application, indicating that the stress pathway may be involved in the development of NMDA-induced spasms. Other brain regions where c-fos was upregulated are MO, SS, VIC, CP, and GPs. These areas are involved in the sensorimotor neural circuits (orange in Figure 6E). It may be possible that sensorimotor circuits are activated in response to the body movement during seizures. Yet another mechanism may not be excluded.

Neuroinflammation in the brain is frequently associated with epilepsy. The proliferation of astrocytes and microglia is a common sign for neuroinflammation. Since astrocytes develop in later developmental stages, proliferation of microglia is more dominant in neuroinflammation in juvenile mice. Unexpectedly, we failed to detect the proliferation of microglia in the juvenile mouse brains with NMDA-induced seizure. One possibility may be that P13 is too early to induce microglial proliferation.

ACTH was ineffective in NMDA-induced seizures in SIK1-MT mice. Although this mechanism is elusive, the interplay between SIK1 and ACTH signaling may provide a clue to explain the result. ACTH administration increased SIK1 gene expression in one hour in our study, which is consistent with a previous report [2]. SIK1 is a phosphorylation target for PKA that is activated by ACTH through the G protein-coupled melanocortin receptor 2. Once SIK1 is phosphorylated by PKA, the SIK1 protein is translocated from the nucleus to the cytosol. Translocation of SIK1 has been suggested to regulate the gene expression mediated by MEF2. In the nucleus, transcriptional regulatory factor MEF2 function is repressed by binding with type II HDAC. SIK1 phosphorylates type II HDAC and removes it from MEF2. The C-terminal truncated form of SIK1 is unable to reside in the nucleus, therefore, it cannot activate the MEF2-mediated target gene transcription [14,15,25,32]. Cytosolic SIK1 phosphorylates CRTC, a coactivator for CREB [7,30,31]. ACTH may control the balance between MEF2 and CREB-mediated gene transcription by regulating the translocation of SIK1. This ACTH-mediated mechanism may be disrupted in the C-terminal truncated form of SIK1.

Our extensive study identified that activated neurons in response to PTZ- and NMDA-induced seizures are distinctive within the layer formation in the somatosensory cortex. PTZ induces acute seizures by blocking inhibitory neurotransmission by binding to the GABA_A_ receptor [33]. NMDA activates neurons through NMDA receptors. The NMDA receptor consists of two GluN1 and two GluN2 subunits. In the cerebral cortex, thalamus, and hypothalamus, the GluN2B subunit is predominantly expressed in early neural development, whereas the expression of the GluN2A subunit begins at two to three weeks of age in mice [34]. Therefore, the seizures induced by NMDA in our study may be caused by the GluN2B-containing NMDA receptor. Multiple mutations of Grin2b, the gene that encodes GluN2B, were identified in patients with infantile spasms, and Grin2b is also classified as the causative gene for DEE-27 [35,36]. The distinct activation patterns of the brain regions between PTZ- and NMDA-induced seizures may be attributable to the pharmacological effect of these drugs on epileptogenesis. Feldman et al. reported that the levels of SIK1 mRNA in the rat hippocampus and cortex were elevated up to eightfold one hour after kainic-acid-induced seizures in male rats [11]. SIK1, as well as its downstream genes, such as CREB and MEF2, are involved in activity dependent neuronal plasticity to reconstruct neuronal circuits. Further studies are required to answer the question of whether the mesoscopic structural change of neuronal circuits is responsible for the increased susceptivity of epileptic seizures by the mutation of the SIK1 gene.

## 4. Materials and Methods

### 4.1. Animals

All procedures of animal experiments were reviewed by the Committee for Animal Experiments of Shinshu University and approved by the president of Shinshu University. Mice were group-housed under environmentally controlled conditions (12:12 light:dark cycle, 22 ± 2 °C, and 55 ± 10% relative humidity) with food and water ad libitum.

The establishment of SIK1-MT mice was described in our previous research [15]. Briefly, we prepared and electroporated mRNAs of Cas9 and sgRNA, targeting the mouse SIK1 gene to the fertilized eggs from C57BL/6JmsSlc mice (Japan SLC Inc., Hamamatsu, Shizuoka, Japan) by following the TAKE method [37,38,39,40]. A male mouse having 8 bp deletion that caused a frameshift in the C-terminal region of the SIK1 protein was screened and backcrossed with C57Bl/6J mice for more than ten generations. The genotyping of the mice was performed by genomic PCR using primer pairs (Forward: 5′-CCACATGGCAGGACACATCT-3′ and Reverse:5-TAAACCCCTGCCTGCTCTTG-3′), followed by the digestion of unmatched PCR products by T7 endonuclease I (New England Biolabs, Ipswich, MA, USA).

To investigate the survival of the mice, we counted the number of pups at the date of birth (P0), P1, P3, P5, P7, P10, P14, P21, P28, P35 (the date of weaning), and P42.

### 4.2. NMDA-Induced Spasms and ACTH Treatment

To induce spasms in mice, we used the NMDA induction method, originally developed in mice by Shi et al. [23]. Postnatal 13-day-old male mice were injected with saline or NMDA in saline at a concentration of 10 or 5 mg/kg body weight [20,23,24]. The onsets and the numbers of tail flickerings and series of body hyperflexions of mice were observed and counted within 40 min after the injection of NMDA or saline. In ACTH treatment experiments, a synthetic analog of ACTH (Cortrosyn Z, 100 U/kg) was injected 1 h before the NMDA injection.

### 4.3. Electrophysiological Ananysis

A patch clamp recording was performed in the same way we used previously [15]. Brains from 13-day-old male mice were cut into 350 μm-thickness in ice-cold-slicing artificial corticospinal fluid (ACSF, in mM: 85 NaCl, 75 sucrose, 2.5 KCl, 1.25 NaH_2_PO_4_, 24 NaHCO_3_, 25 glucose, 0.5 CaCl_2_, and 4 MgCl_2_) saturated with 95% O_2_/5% CO_2_. Brain slices were transferred to a recovery chamber filled with recording ACSF (in mM: 126 NaCl, 2.5 KCl, 1.25 NaH_2_PO_4_, 26 NaHCO_3_, 10 glucose, 2 CaCl_2_, and 2 MgCl_2_), followed by incubating at 32 °C for 30 min, and then at room temperature for 30 min. In the current-clamp experiments, glass pipettes (4–8 M ohm) filled with a potassium-based intra-cellular solution (ICS, in mM: 130 K Gluconate, 6 KCl, 10 HEPES, 1 EGTA, 2.5 MgCl_2_, 2 magnesium ATP, 0.5 sodium GTP, 10 phosphocreatine sodium, 290 mOsm) were used. The resting membrane potential was measured immediately after establishing a whole-cell recording. Hyperpolarizing and depolarizing step pulses (700 ms) were applied to characterize the neuronal firing property. The membrane potential at which the temporal rate of the potential reached 10 mV/ms was defined as the action potential threshold.

### 4.4. Pentylenetetrazol-Induced Seizure

Six-week-old mice were injected with pentylenetetrazol (PTZ) in saline (50 mg/kg, i.p.). The development of the PTZ-induced seizure was scored based on a modified Racine’s scale [41,42]: (0) no abnormal movements; (1) reduced motility and prostate position; (2) partial clonus; (3) generalized clonus including extremities; (4) tonic-clonic seizure with rigid paw extension; (5) death. The score was determined every minute during a 10-min observation.

### 4.5. Histological Analysis

Histological analysis was followed by the previous procedures [43,44,45]. Briefly, under deep anesthesia, the mice were perfused transcardially with ice-cold phosphate-buffered saline (PBS, pH 7.4), followed by 4% paraformaldehyde in PBS. Fifty-μm-thick coronal sections were prepared with a sliding microtome (REM-700, Yamato Kohki Industrial, Asaka, Saitama, Japan). The sections were washed with PBS, blocked with PBS containing 1% bovine serum albumin, 0.1% Triton-X100, and 10% of normal donkey serum and incubated with rabbit anti-c-fos (1:2000, Abcam, Cambridge, UK), rabbit anti-GFAP (1:200, DAKO, Glostrup Kommune, Denmark), or rabbit anti-Iba1 antibody (1:200, Wako, Osaka, Japan). After an overnight incubation with the primary antibodies, the brain sections were washed with PBS containing 0.1% Triton-X100 and incubated with Cy2-conjugated donkey antibody against rabbit IgG (1:400, Jackson immunoresearch, West Grove, PA, USA), respectively, for 2–3 h at room temperature. After further washing with PBS, the brain sections were mounted on a slide glass, counterstained with DAPI, and coverslipped. Fluorescence images were taken with an all-in-one fluorescent microscope (BZ-X710 and BZ-X810, Keyence, Osaka, Japan) and confocal laser-scanning microscopes (TCS SP8; Leica Microsystems, Wetzlar, Germany). For quantitative analysis of c-fos-expressing cells, TIFF images of coronal brain slices were aligned to the Allen Mouse Brain Common Coordinate Framework version 3 (ABA) on ImageJ (version 2.3.0, FIJI). The c-fos positive cells were defined using the signal intensity and counted on ImageJ.

### 4.6. Quantification of SIK1 mRNA in the Brain

The expression level of Sik1 was quantified by qPCR using GAPDH as an endogenous control. In short, six of the 13-day-old mice were treated with ACTH (100 U/kg), and five mice were treated with saline as the controls. Tissues were quickly removed, trimmed and frozen. Total RNA was extracted from frozen tissue using RNAiso (Takara), following the manufacturer’s instruction. A total of 1 ug RNA was converted to cDNA using the High Capacity cDNA Reverse Transcription Kit (Applied Biosystems, Waltham, MA, USA). An amount of 50 ng cDNA was subjected to qPCR using Power SYBR Green PCR Mix (Applied Biosystems) on QuantStudio3 (Thermo Fisher, Waltham, MA, USA). qPCR was replicated three times for each sample. We used a primer set for SIK1 (Forward: 5′-GACGGAGAGCGTCTGATACC-3′ and Reverse: 5-GAGCCAACCCTTTGATCTTG-3′) [12] and one for GAPDH (Forward: 5′-CATGGCCTTCCGTGTTCCTA-3′ and Reverse: 5-CCTGCTTCACCACCTTCTTGA-3′). The fold changes of SIK1 mRNA were calculated by the delta-delta Ct method.

### 4.7. Sample Size and Statistical Analysis

Samples sizes were determined based on the established practice and our previous experience in the respective assays. The number of independent samples (e.g., neurons) is indicated on the graphs, and the numbers of animals are indicated in the figure legends. All values represent the average of independent experiments ± SEM. The variance among the analyzed samples was similar. Statistical significance was determined by parametric Student’s *t*-test, nonparametric Wilcoxon rank-sum test or ANOVA followed by Bonferroni post hoc test. For comparison of the survival curves, we used log-rank (Mantel-Cox) and Gehan–Breslow–Wilcoxon tests. Statistical analysis was performed by custom-written R scripts or Prism 8.4.3 (Graphpad Software Inc., San Diego, CA, USA). Statistical significance is indicated by asterisks (* *p* < 0.05, ** *p* < 0.01, *** *p* < 0.001). All data are expressed as means ± SEM.

## 5. Conclusions

Epilepsy-associated SIK1 mutant mice were subjected to NMDA- and PTZ-induced seizures, rodent models for infantile spasms, and temporal lobe epilepsy. The seizures became more severe in the SIK1-MT mice than in the wild-type mice. ACTH treatment failed to mitigate the severity of NMDA-induced spasms in SIK1-MT mice. As suggested in human clinical research, our data support the hypothesis that the SIK1 gene is closely associated with epileptic seizures and raise the notion that SIK1 may be involved in the molecular pathway underlying the ACTH therapy for infantile spasms.

## Data Availability

All datasets used and/or analyzed during the current study are available from the corresponding authors on reasonable request.

## Supplementary Materials

The following supporting information can be downloaded at: https://www.mdpi.com/article/10.3390/ijms23147927/s1.
