# Supplementary figures and images for: An Epilepsy-Associated Mutation of Salt-Inducible Kinase 1 Increases the Susceptibility to Epileptic Seizures and Interferes with Adrenocorticotropic Hormone Therapy for Infantile Spasms in Mice
Source: Int J Mol Sci. 2022 Jul 18;23(14):7927. doi: 10.3390/ijms23147927 (PMC9319016; doi:10.3390/ijms23147927)

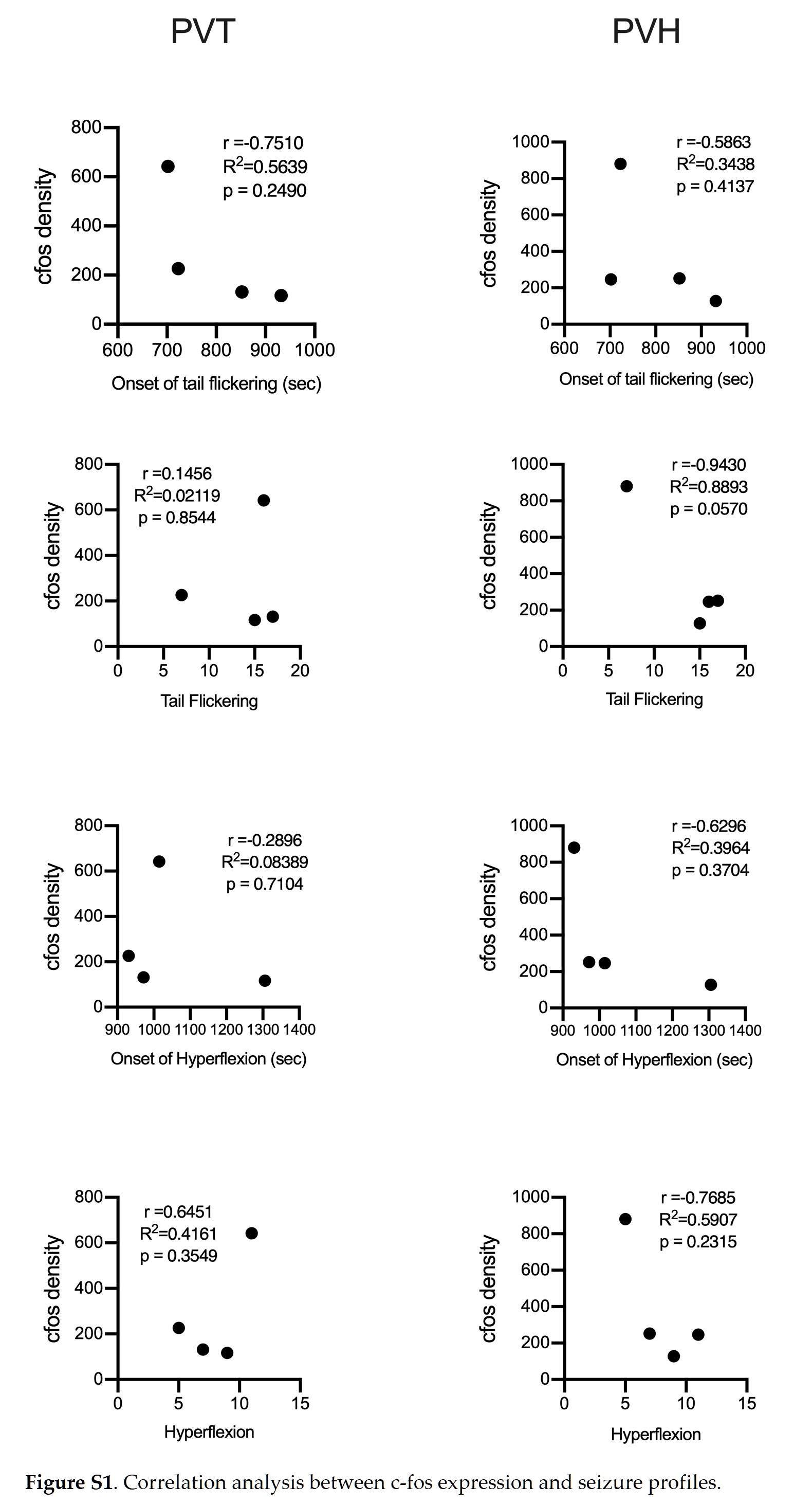

Supplement: Supplementary file 1 [file ijms-23-07927-s001.zip › ijms-1809300-supplementary-2/Supplementary Figure S1-Correlation analysis between c-fos expression and seizure profiles.jpg]
